# Effectiveness of pharmacovigilance: multifaceted educational intervention related to the knowledge, skills and attitudes of multidisciplinary hospital staff

**DOI:** 10.6061/clinics/2017(01)09

**Published:** 2017-01

**Authors:** Fabiana Rossi Varallo, Cleopatra S Planeta, Patricia de Carvalho Mastroianni

**Affiliations:** I Universidade Estadual Paulista – UNESP, Departamento de Fármacos e Medicamentos, Araraquara/SP, Brazil; II Ministério da Educação do Brasil, Fundação CAPES, Brasília/DF, Brazil; III Universidade Estadual Paulista – UNESP, Faculdade de Ciências Farmacêuticas, Laboratório de Farmacologia, Araraquara/SP, Brazil

**Keywords:** Pharmacovigilance, Health Knowledge, Attitudes, Practices, Interventional Studies, Product Surveillance

## Abstract

**OBJECTIVES:**

Most educational interventions in pharmacovigilance are designed to encourage physicians to report adverse drug reactions. However, multidisciplinary teams may play an important role in reporting drug-related problems. This study assessed the impact of a multifaceted educational intervention in pharmacovigilance on the knowledge, skills and attitudes of hospital professionals.

**METHOD:**

This prospective, open-label, non-randomized study was performed in a medium-complexity hospital in São Paulo, Brazil. The intervention involved four activities: 1) an interactive lecture, 2) a practical class, 3) a pre-post questionnaire administered to professionals on a multidisciplinary team, and 4) educational material. The intervention’s impact on the professionals’ knowledge and skills was assessed using the World Health Organization’s definitions. The intervention’s effect on the professionals’ attitudes was analysed by the prevalence of adverse drug event reports (adverse drug reactions, medication errors, therapeutic failure and drug quality deviations) and the relevance (seriousness and expectancy) of the events.

**RESULTS:**

One hundred seventy-three professionals were enrolled. A 70-fold increase in the number of adverse drug event reports was observed during the 12 months post-intervention. The intervention improved the professionals’ form-completion skills (*p*<0.0001) and their knowledge of pharmacovigilance (*p*<0.0001). The intervention also contributed to detecting serious drug-induced events. The nursing staff reported medication errors, and pharmacists and physiotherapists recognized serious adverse drug reactions. Physicians communicated suspicions of therapeutic failure.

**CONCLUSIONS:**

A multidisciplinary approach to drug-safety assessments contributes to identifying new, relevant drug-related problems and improving the rate of adverse drug event reporting. This strategy may therefore be applied to improve risk communication in hospitals.

## INTRODUCTION

Although the limitations of traditional systems designed to detect adverse drug events (ADEs) are well known 1, spontaneous reporting remains the cornerstone of post-marketing drug safety surveillance 1,2.

The use of spontaneous reporting persists because new approaches that have been developed to improve risk communication (e.g., electronic health record databases) may not have enough exposure to new drugs 3. Consequently, spontaneous reporting should continue to be used in phase IV studies to provide information regarding the safety and effectiveness of drug use 3 and to detect unexpected and serious events 4.

There are a limited number of professionals with knowledge of pharmacovigilance around the world 5. However, the number of health employees with competencies related to drug surveillance in low- and middle-income countries is very low, as there are often limited financial resources available in these countries to support attendance at professional development courses in pharmacovigilance 5. Consequently, there are a limited number of professionals in developing countries capable of performing drug safety assessments 5-7 and improving risk management.

Although the contributions of Latin American countries to the field of pharmacovigilance has increased in recent years 8, a minority of Latin American nations invest in professional training 9-10. Therefore, biases in epidemiological communication 11 and impairments in signal detection result from the lack of robust information available during the post-marketing life cycle of drugs.

Several strategies have been implemented to promote adherence to appropriate pharmacovigilance practices among health professionals in pharmacovigilance services, and these have primarily involved designing educational interventions 12-14, including subjects related to drug safety assessments in both undergraduate and postgraduate programs 15, and increasing the amount of time dedicated to teaching pharmacovigilance in universities 16. The most important objective of these educational interventions is to develop functional and behavioural competencies 17 to promote changes in attitude among physicians and pharmacists towards adverse drug reaction (ADR) reporting. These educational interventions do not include other types of ADEs, such as medication errors, therapeutic failure and drug quality deviations. Consequently, drug-induced harm is underreported.

Given the constraints of healthcare assistance in low- and middle-income countries (brief contact between physicians and patients, lack of time to report drug-related problems and lack of information in patients’ records about drug safety), the responsibility for ADEs must be shared among trained healthcare professionals (pharmacists, nurses) 5 to decrease underreporting 10. However, studies of multidisciplinary teams are scarce in the literature.

Based on these considerations, the current study aimed to 1) assess the impact of a multifaceted educational intervention (MEI) on the prevalence of ADE reporting (attitude) by multidisciplinary teams, 2) evaluate the effectiveness of the MEI on professionals’ knowledge of and skills in pharmacovigilance and their fulfilment of ADE reporting requirements, and 3) assess the degree of relevance of the ADE reports submitted after the MEI according to the events’ seriousness and likelihood of occurrence.

## MATERIALS AND METHODS

### Study population and setting

The study was conducted in a medium-complexity, general and public hospital with 94 beds. The hospital is located in the Araraquara-São Carlos region (São Paulo State, Brazil), which comprises 24 municipalities and includes over one million inhabitants. The hospital is known for providing healthcare for infectious diseases and neurologic rehabilitation (approximately 13,000 appointments per month).

The inclusion criteria comprised all hospital staff (physicians, pharmacists, nurses, auxiliary nursing and pharmacy staff, and administrative officers, as well as social assistants, physiotherapists, audiologists, nutritionists, psychologists, and occupational therapists) with an employment link to the hospital who agreed to participate by signing an informed consent form. The exclusion criteria included professionals who were on sick leave or vacation, those who were not eligible to participate in the intervention and those who declined to respond to the questionnaire despite having shown interest in the intervention by participating in the lecture and practical class.

### Design of the study

A longitudinal, prospective, open-label and non-randomized study using a single group before-and-after intervention design 18 was conducted from 1 February 2012 through 31 May 2012. A before-and-after design measures the performance of one or more subjects after the introduction of an intervention to assess the intervention’s effect 19.

Although randomized controlled trials represent the most suitable research design for evaluating the effectiveness of an intervention, 20 we were unable to design a randomized controlled trial due to the unavailability of an appropriate control group. Therefore, we opted to use the before-and-after design due to its simplicity and its robustness relative to observational research 19. Furthermore, findings in the literature have demonstrated the positive impact of educational interventions in pharmacovigilance on changing the behaviour of health professionals 12,13. Additionally, because the introduction of an intervention may improve risk communication, we decided that ethical principles could not be satisfied if a control group was unable to benefit from the acquisition of knowledge regarding drug safety assessments through the intervention.

### Multifaceted educational intervention

The applied MEI model was first proposed by Pagotto et al. (2013) 12. To assess the reproducibility of the method, a pilot study was conducted in six hospital institutions in Mercosur member countries 10. Briefly, the MEI involved four different activities: a lecture on pharmacovigilance concepts and the landscape of pharmacovigilance; a practical class on how to correctly complete ADE reports; the distribution of educational materials and the administration of a questionnaire to the participating professionals before and after the MEI. These activities were conducted during four separate sessions, each of which lasted one hour.

In the first session, the pre-test assessment was administered via questionnaire. The instrument’s development and the determination of its concepts and constructs of interest were based on the literature 10. The questionnaire included three sections: the first section related to knowledge assessment (five essay questions about the importance of pharmacovigilance), the second section focused on attitudes (five essay questions related to ADE reporting) and the third section consisted of an evaluation of the professional’s skills regarding information that is considered essential, necessary and unnecessary on an ADE report. This portion of the study was designed to obtain baseline data about the hospital staff members’ knowledge, skills and attitudes related to pharmacovigilance.

The second and third sessions involved lectures, a practical class, and the distribution of educational material to multidisciplinary teams of hospital staff. The post-test assessment was conducted during the final session. The participants were asked to fill out the same questionnaire they completed in the first session to enable an evaluation the effectiveness of the intervention on their knowledge, skills and attitudes 10.

### Follow up and outcome measures

The knowledge assessment was performed by analysing the responses to the questionnaire; each item on the questionnaire was assigned a score ranging from 0–10. The World Health Organization’s definitions for pharmacovigilance 21 were considered gold-standard answers (Table 1). Scores below 5 were considered unsatisfactory, scores between 5 and 7.5 were considered average and scores above 7.6 were considered satisfactory in terms of knowledge acquisition.

The skills evaluation was based on the professionals’ perception of the degree of relevance of the information reported on an ADE form. Accordingly, the professionals were asked to highlight the ADE form fields according to whether the data required for those fields are considered unnecessary, necessary or essential. The minimal and desirable criteria to be supplied in an ADE form were defined according to the recommendations of the Pan-American Health Organization 22 (Table 1). The Wilcoxon-Mann-Whitney statistical test was used to assess the impact of the MEI on the participants’ knowledge and skills. Statistical significance was set at p<0.05.

To analyse the impact of the MEI on the attitudes and behaviour of the participating professionals, the absolute number of ADE reports (ADR, therapeutic failure, medication errors and quality deviations) 12 months prior to the intervention (t_0_) were investigated. A year of follow up regarding the absolute number of ADEs reported post-intervention was also performed (t_2_) to assess changes relative to t_0_.

The Mann-Whitney statistical test was used to identify significant differences between the proportions of the number of ADE reports/the number of hospitalized patients before the educational intervention (t_0_) and after the educational intervention (t_2_). An evaluation of the effectiveness of the MEI on ADE reporting was performed using segmented generalized linear regression models. Adjustment segmentation was conducted by including a variable that represented the period over which the MEI occurred (t_1_).

The relevance of the ADR and medication error reports made during the study period was also evaluated (seriousness and expectancy of occurrence). The seriousness of medication errors was assessed according to the standards of the National Coordinating Council for Medication Error Reporting and Prevention 23. Serious ADRs were defined as those causing hospitalization, those that were fatal or life-threatening, or those that resulted in significant changes in patient treatment (thereby prolonging hospitalization) 24. Informational drug sheets approved by the National Agency of Sanitary Surveillance (ANVISA) and monographs, such as those in the DRUGDEX (MICROMEDEX^®^database), Uptodate^®^ database and LexiComp Manole (2009), were consulted to verify the expectancy of an ADR.

### Approval by the ethics committee and clinical trial registration

The study (protocol E-015/10) was approved by the Ethics Committee in Research of the Instituto Lauro de Souza Lima. The protocol for the research was registered (identification number: NCT02134587).

## RESULTS

During the study, the staff of the hospital included 421 professionals, of whom 334 (79.3%) satisfied the inclusion criteria. Of these 334 individuals, 203 (60.8%) returned the questionnaire; however, only 173 staff members (51.7%) completed the intervention. One hundred thirty-one subjects (48.2%) attended at least one of the intervention sessions.

The MEI was effective at increasing knowledge in pharmacovigilance among the 173 professionals who participated in all the sessions (*p*<0.0001). Prior to the intervention, the professionals’ knowledge was classified as unsatisfactory, whereas post-intervention it was classified as average. The MEI also improved the professionals’ skills in filling out ADE forms (*p*<0.0001) (Table 2), mainly among the physicians, nurses and pharmacy auxiliaries (Table 2). The skills of the other professionals remained at the average level (Table 2).

Prior to the MEI (t_0_), only three ADE reports had been submitted, all of which related to therapeutic failure. After 12 months of follow up, 215 ADEs were reported. Of these ADEs, 166 corresponded to medication errors, 26 corresponded to ADRs, 18 corresponded to quality deviations and 5 corresponded to therapeutic failures. Therefore, the MEI increased the absolute number of ADE reports by 70-fold.

**Figure 1:**
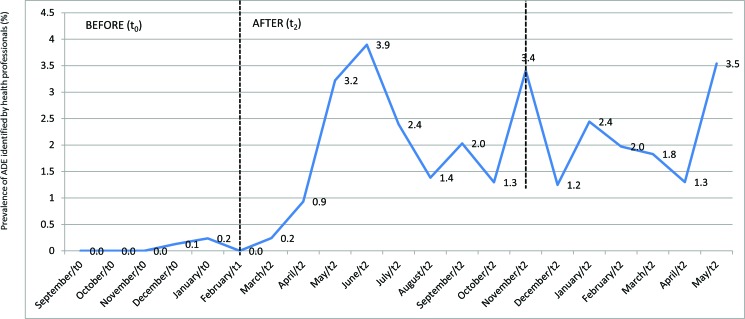
Adverse drug event prevalence detected via voluntary reporting by professionals before (t_0_) and after the educational intervention (t_2_) in a public and general hospital. Am rico Brasiliense-SP (Brazil).

The prevalence of major ADEs reported prior to the MEI (t_0_) was 0.2%, and the prevalence increased to 3.9% after the MEI (t2) (Figure 1). According to the Mann-Whitney test, there was a significant difference between the proportion of ADE reports/inpatients following the educational intervention (*p*=0.003). The data suggest that the MEI promoted changes in the participants’ behaviours and attitudes related to ADE reporting. According to the segmented generalized linear regression model, the MEI significantly contributed to the increase in the number of ADE reports (*p*=0.0005).

Regarding the period of effectiveness of the MEI, a decrease of 2.5% (3.9% compared with 1.4%) was noted in the prevalence of ADEs reported four months after the MEI was implemented (Figure 1). However, the ADE reporting rate did not return to its pre-intervention level. Subsequent increases in the prevalence of ADE reporting (3.4% and 3.5%) (Figure 1) occurred due to the implementation of policies regarding risk management and patient safety at the institution.

Serious ADRs were reported by pharmacists (N=6) and physiotherapists (N=1). The nursing staff submitted the most ADE reports (150), followed by pharmacists (N=29), physicians (N=6) and auxiliary nursing staff members (N=3). The professional classification of twenty-six professionals who reported ADEs could not be determined. Medication errors (N=136) and quality deviations (N=10) were often reported by the nursing staff, whereas ADRs (N=14) were reported by pharmacists, and therapeutic failures (N=5) were reported by physicians.

**Figure 2:**
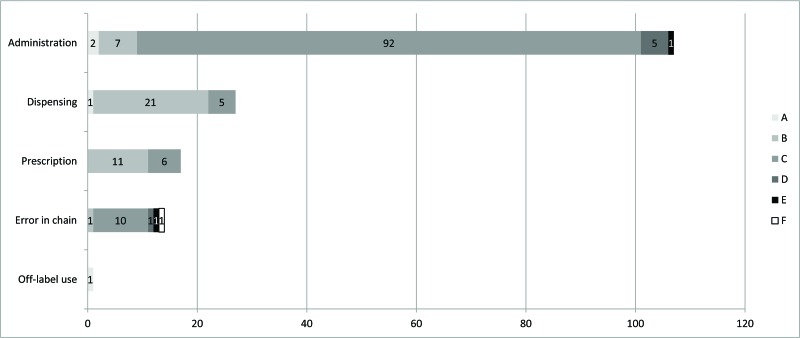
Absolute number of medication error reports made by professionals after the educational intervention (t_2_) (no reports of this type were made prior to the research) according to aetiology and seriousness (N=166). Am rico Brasiliense-SP (Brazil).

A broad spectrum of medication errors were reported after the MEI, whereas no ADEs related to medication errors were reported prior to the MEI (Figure 2). Administration errors were the most frequently identified errors (Figure 2), and these related mainly to the delayed intake of a drug (N=23).

Two of the 26 ADR reports were of poor quality, as they lacked information regarding the suspicious drug associated with the event and the time of the onset of the event. The remaining 24 ADR reports were well documented, which enabled the detection of 29 clinical manifestations likely induced by 19 different drugs. With the exception of four events, whose likelihood of being drug-related was remote (alternative causes may explain these events), all the events were expected ADRs. Seven of these were considered serious for the following reasons: increased hospital stay (N=4), temporary disability (N=2) and induced hospitalization (N=1).

## DISCUSSION

This study indicates that using MEIs with multidisciplinary teams has a positive effect on the awareness of hospital staff with respect to the importance of ADE reporting. An increase exceeding 100% in the absolute number of reports of drug-induced events was observed in the current research. The strategies applied in the intervention improved the participants’ knowledge of pharmacovigilance and increased their skills in correctly filling out report forms, primarily in relation to medication errors. Therefore, including hospital professionals in pharmacovigilance services may contribute to patient safety 25 by stimulating the detection of harmful drug-induced events and the development of strategies designed to prevent such events.

In recent years, legislation regarding pharmacovigilance has been modified and updated to widen the scope of post-marketing surveillance and improve individual patient care 26. The primary goal of efforts to increase communication about drug-related problems is to facilitate medication error reporting and subsequently learn from those errors in order to contribute to the safe and effective use of medicines for the benefit of patients and public health 27.

To ensure a safe environment for drug therapy during all stages of a medicinal product’s life cycle, it is necessary to increase the involvement of healthcare professionals in pharmacovigilance 5.

A systematic review suggests that interprofessional collaborations promote benefits to healthcare assistance and improve patient outcomes 28. Engaging a multidisciplinary team in an educational intervention for pharmacovigilance revealed a remarkable increase in the prevalence of medication error reporting, mainly by the nursing staff. Moreover, the study observed drug-induced events associated with harm to the patient that went unreported prior to the MEI.

Therefore, our findings highlight the importance of adopting a multidisciplinary perspective to address the importance of pharmacovigilance in contributing to patient safety and enhancing the quality of reports related to medication errors 29. Recognizing events that could potentially be prevented is fundamental to identifying weaknesses in risk management procedures 30 and designing policies to improve safety protocols.

In this study, physiotherapists reported serious ADRs, corroborating the interdisciplinary nature of pharmacovigilance activities and their relation to patient safety 31, since these professionals are also able to recognize idiosyncratic events 32,33. Therefore, physiotherapists play an important role in contributing to drug safety analysis and to the prevention, early detection and communication of ADEs 33. Furthermore, clinical pharmacists follow up on the safety of pharmacotherapy and can contribute to improving the early detection of drug-induced harm. Physicians are focused on patient diagnoses; therefore, monitoring the effectiveness of a prescribed treatment is fundamental to assessing improvements in clinical outcomes and determining the target for the best pharmacotherapy.

Although nutritionists, psychologists, occupational therapists and administrative officers did not submit any ADE reports following the MEI, these professionals described suspicious events in patient records. While these drug-related problems were not appropriately communicated through ADE reports, there was an improvement in risk communication in patient charts.

Even though a substantial fraction of the hospital’s professionals did not complete the four sessions (i.e., a low rate of return for the questionnaire), we can conclude that the MEI related to pharmacovigilance instilled a culture of drug safety at our institution. Our hypothesis was consubstantiated by the higher-quality information included in patient records. Irrespective of whether professionals formally reported ADEs to the risk management team at the hospital, they were able to enhance the ADE reporting rate by 10% (data not shown).

Regarding the period of the MEI’s effectiveness, the number of ADE reports decreased after the first four months, and the same behaviour was noted in the literature 2,34. The period during which the rate of ADE reporting drops after educational interventions depends on the techniques applied in the intervention, the level of healthcare and the professionals enrolled in the intervention 34-35. Therefore, the data suggest the need for periodic educational interventions to maintain motivation among professionals regarding ADE reporting.

It is important to highlight that the subsequent increases observed in ADE reporting coincided with the implementation of patient safety policies and the deployment of a hospital risk management team. Therefore, both strategies (risk-management policies and educational interventions) are inextricably linked in efforts to follow up on drug safety and treatment effectiveness in hospitals.

The tradition of pharmacovigilance is a recent arrival in Latin America, and there are few publications related to pharmacovigilance 2. Additional studies are necessary to assess the impact of ADE in low- and middle-income countries 5. Consequently, our educational initiative is relevant to efforts endeavouring to motivate volunteers to increase post-marketing surveillance, decrease underreporting, address the new trends derived from legislation and contribute to learning about ADE.

### Limitations of the study

The findings of this study should be considered in the context of the study’s two primary design limitations. First, randomized controlled trials are considered the gold standard for evaluating the effectiveness of an intervention. However, before-and-after intervention designs are ideal if conducting a randomized controlled trial is either logistically or ethically not possible. Second, during the follow-up period for the MEI’s effectiveness, a policy regarding risk management and patient safety was deployed in our hospital. However, the implementation of this policy did not involve providing hospital staff members with training related to pharmacovigilance activities.

The MEI implemented for hospital staff in this study improved the relevance of ADE reports and increased the prevalence of such reports. Furthermore, the MEI was effective at enhancing awareness (knowledge) of pharmacovigilance, even among individuals who did not complete all the intervention protocols or return the questionnaire. This MEI can motivate the members of a multidisciplinary team to change their behaviours/attitudes and can contribute to improving the team members’ skills in detecting new and relevant ADEs. Therefore, the inclusion of hospital staff in drug safety analysis is an important strategy to decrease ADE underreporting and improve the communication risks associated with drug use.

## AUTHOR CONTRIBUTIONS

Varallo FR, Planeta CS and Mastroianni PC conceived and designed the study and were responsible for the data analysis and interpretation, and manuscript writing. All of the authors approved the final version of the manuscript.

## ACKNOWLEDGMENTS

We thank the CAPES Foundation, Ministry of Education of Brazil for the scholarship (PDSE) grant n°. 014301/2013-00. The authors would also like to thank the São Paulo Research Foundation (FAPESP) for providing the financial support for this project under grant #2013/10263-9 and Programa de Apoio ao Desenvolvimento Científico da Faculdade de Ciências Farmacêuticas da UNESP-PADC. We are also grateful to the Hospital Estadual Américo Brasiliense for allowing us to collect the study data.

## Figures and Tables

**Table 1:** Criteria considered to be gold standard in terms of knowledge [32] and skill [2] assessment in pharmacovigilance.

KNOWLEDGE	GOLD-STANDARD ANSWERS	SKILLS	GOLD-STANDARD ANSWERS
1) What is pharmacovigilance?	The monitoring of medicine use for the detection, assessment and prevention of adverse drug reactions and any issues related to medicine.	1) ADR reporting 1.1) Minimal criteria	About the patient: name, medical record number, bed, gender, clinical history (illness, comorbidities, drug allergies).
2) Does the practice of pharmacovigilance promote benefits? When positive, what are the benefits and who are the beneficiaries?	The practice of pharmacovigilance promotes benefits for drug users, professionals and healthcare institutions. The benefits include contributing to patient safety, improving the quality of care in health facilities, and ensuring that drugs on the pharmaceutical market are safe, effective and high quality.		About ADR: clinical manifestations (treatment initiation and finalization), evolution (outcomes), laboratory tests, treatment (hospitalization, discontinuation of drugs, prescription of drugs). About the suspected drug: drug, dose, route of administration, indication, date of treatment initiation and finalization, expiry date, lot number and manufacturer.
3) Who may notify?	Drug users, health professionals and the pharmaceutical industry.		About polypharmacy: drugs, dose, route of administration, date of treatment initiation and finalization, expiry date, lot number and manufacturer.
4) What can you notify?	Any drug-related problems, especially adverse drug reactions, medication errors, therapeutic ineffectiveness and drug-quality deviations.		About the reporter: name, profession, telephone number or e-mail and date that the report was made.
5) What do you mean by:		1.2) Desirable criteria	About the patient: risk factors for ADR occurrence (kidney and/or hepatic failure, previous exposure to the drug, alcohol consumption or a smoking habit).
a) Adverse drug event?	Any damage or harm caused to patients arising from drug use.		
b) Adverse drug reaction?	A response to a drug that is noxious and unintended and that occurs at doses normally used in humans for prophylaxis, diagnosis, therapy of disease, or for modifications to physiological function.		About the ADR: clinical evolution (outcomes), documentation of diagnoses (including procedures applied, data from laboratory tests and pharmacological treatment).
c) Medication errors?	Any preventable event that may cause or lead to inappropriate medication use or patient harm while the medication is in the control of the healthcare professional, patient, or consumer.	2) Medication error reporting	
d) Quality drug deviations?	A departure from the quality parameters established for a product or process. In pharmacovigilance, these deviations can include organoleptic changes that are either physico-chemical and/or general (leaks, inadequate labelling, foreign particles, etc.).	2.1) Desirable criteria	About the product: the product involved in the error (dose, route of administration, expiry date, lot number, type of packaging, label, etc.).
e) Suspicious of therapeutic ineffectiveness?	The total or partial absence of the expected effect of the drug on the condition of use prescribed or indicated in the leaflet.		About the place: where the error occurred (appropriateness to carrying out the activities, promotes distractions, considerable noise, etc.), medical equipment and products used (meet the attributes of quality and safety), service flow, etc.
6) What is the correlation between pharmacovigilance and drug safety?	The practice of pharmacovigilance, monitoring drug use, contributes to the regulation of the pharmaceutical market because it focuses on the safety, quality and effectiveness of these products.		About the person involved: characterize regarding time of graduation, time of employment, duty period, aetiologic causes and contributing factors.
7) How would you explain why a drug does not produce the desired effect?	The medicine may not produce the desired effect for three primary reasons: the inherent characteristics of the patient, medication errors and quality deviation.		
8) In which stages of drug use can medication errors occur?	In all stages: prescribing, dispensing and administration.		

**Table 2:** Adherence of hospital staff and the impact of educational interventions on knowledge related to pharmacovigilance and skill filling out forms related to adverse drug events according to professional classification (N=173). Américo Brasiliense-SP (Brazil).

Professional	Adherence			Impact					
	Answered the questionnaire (N)	Number employed (N)	Return rate (N)	Knowledge			Skills		
				Before Mi (min-max)	After Mi (min-max)	*p*-value	Before Mi (min-max)	After Mi (min-max)	*p*-value
Physicians	11	86	12.8	4.3 (2.9-6.6)	5.0 (0.4-9.3)	0.006*	4.0 (0.0-7.9)	5.2 (0.0-9.2)	0.007*
*Multidisciplinary team*									
Social assistant	4	5	80.0	3.9 (1.3-5.4)	6.3 (4.8-9.3)	<0.0001*	5.2 (3.8-6.2)	6.1 (4.7-7.9)	<0.001*
Pharmacist	1	2	50.0	4.8 (NA)	5.9 (NA)		0.0 (NA)	0.0 (NA)	
Physiotherapist	10	12	83.3	2.1 (1.0-6.0)	5.8 (4.3-9.2)		5.4 (3.1-7.7)	6.8 (4.5-7.7)	
Audiologist	2	4	50.0	1.2 (0.1-2.4)	6.2 (4.8-7.6)		6.7 (6.2 -7.2)	5.0 (2.9-7.1)	
Nutritionist	5	5	100.0	3.0 (2.0-3.7)	5.8 (4.6-7.4)		5.7 (5.3-6.7)	7.5 (4.7-8.3)	
Psychologist	3	4	75.0	2.7 (2.1-3.4)	6.2 (6.0-7.8)		5.2 (4.9-6.2)	6.1 (4.1-7.7)	
Occupational therapist	4	4	100.0	2.0 (1.4-3.7)	5.5 (3.2-5.7)		4.7 (0.0-6.4)	4.7 (0.0-6.8)	
Auxiliary in pharmacy	9	11	81.8	1.5 (0.0-3.1)	5.9 (4.0-7.1)		2.2 (0.0-6.5)	6.7 (0.0-9.2)	
Administrative officer	5	11	45.4	0.6 (0.0-1.6)	5.8 (3.9-7.0)		5.8 (4.3 -7.0)	5.2 (0.0-6.9)	
*Nursing staff*									
Nurse	26	58	44.8	3.3 (0.7-5.2)	5.5 (2.2-7.7)	<0.0001*	4.9 (0.0-7.2)	6.0 (0.0-8.9)	<0.0001*
Auxiliary nurse	93	219	42.5	2.0 (0.0-5.9)	5.4 (0.9-8.1)		2.0 (0.0-8.0)	4.0 (0.0-9.2)	
TOTAL	173	421		2.3 (0.0-6.6)	5.2 (0.9-9.3)	<0.0001*	3.9 (0.0-8.0)	5.2 (0.0-9.2)	<0.0001*

Note:

Mi=median

Min=minimum

Max=maximum

NA=not applicable.

* Significant value.
